# PTX3 Regulation of Inflammation, Hemostatic Response, Tissue Repair, and Resolution of Fibrosis Favors a Role in Limiting Idiopathic Pulmonary Fibrosis

**DOI:** 10.3389/fimmu.2021.676702

**Published:** 2021-06-21

**Authors:** Andrea Doni, Alberto Mantovani, Barbara Bottazzi, Remo Castro Russo

**Affiliations:** ^1^ Unit of Advanced Optical Microscopy, Department of Immunology and Inflammation, Humanitas Clinical and Research Center IRCCS, Milan, Italy; ^2^ Department of Biomedical Sciences, Humanitas University of Milan, Milan, Italy; ^3^ The William Harvey Research Institute, Queen Mary University of London, London, United Kingdom; ^4^ Laboratory of Pulmonary Immunology and Mechanics, Department of Physiology and Biophysics, Institute of Biological Sciences, Universidade Federal de Minas Gerais, Belo Horizonte, Brazil

**Keywords:** PTX3, inflammation, hemostasis, resolution, fibrosis, IPF, humoral immunity

## Abstract

PTX3 is a soluble pattern recognition molecule (PRM) belonging to the humoral innate immune system, rapidly produced at inflammatory sites by phagocytes and stromal cells in response to infection or tissue injury. PTX3 interacts with microbial moieties and selected pathogens, with molecules of the complement and hemostatic systems, and with extracellular matrix (ECM) components. In wound sites, PTX3 interacts with fibrin and plasminogen and favors a timely removal of fibrin-rich ECM for an efficient tissue repair. Idiopathic Pulmonary Fibrosis (IPF) is a chronic and progressive interstitial lung disease of unknown origin, associated with excessive ECM deposition affecting tissue architecture, with irreversible loss of lung function and impact on the patient’s life quality. Maccarinelli et al. recently demonstrated a protective role of PTX3 using the bleomycin (BLM)-induced experimental model of lung fibrosis, in line with the reported role of PTX3 in tissue repair. However, the mechanisms and therapeutic potential of PTX3 in IPF remained to be investigated. Herein, we provide new insights on the possible role of PTX3 in the development of IPF and BLM-induced lung fibrosis. In mice, PTX3-deficiency was associated with worsening of the disease and with impaired fibrin removal and subsequently increased collagen deposition. In IPF patients, microarray data indicated a down-regulation of PTX3 expression, thus suggesting a potential rational underlying the development of disease. Therefore, we provide new insights for considering PTX3 as a possible target molecule underlying therapeutic intervention in IPF.

## Introduction

### Role of PTX3 in Humoral Innate Immunity

The pentraxin family is an ancient group of evolutionarily conserved proteins belonging to humoral innate immunity that act as pattern recognition molecules (PRM). PTX3, the prototype of the long pentraxins arm, differs from the short pentraxins C reactive protein (CRP) and serum amyloid P component (SAP/PTX2) in molecular structure, gene organization, cellular source, and recognized ligands. PTX3 is rapidly produced by mononuclear phagocytes or stromal cells, including mesenchymal, smooth muscle, and endothelial cells (ECs) (1–5), in response to primary proinflammatory cytokines (IL‐1β and TNF-α), TLR agonists, microbial components (LPS or Outer membrane protein-A) and microbes. The molecule acts as an opsonin during infections, facilitating phagocytosis and activating the complement cascade (6). Genetic evidence in mice (7) and humans (8–15) suggests that PTX3 plays an essential role in resistance against selected pathogens, in particular *A. fumigatus*. In addition, PTX3 is induced in response to tissue injury and, through the interaction with the complement system and ECM components, plays non-redundant roles in tissue repair and cancer (1, 16). The relevance of PTX3 in the assembly of the *cumulus oophorous* was the first evidence of its role in ECM. Female subfertility associated with PTX3-deficiency (2, 6) also emphasizes the importance of this protein in ECM assembly and remodeling.

Inflammation activates various tissue response cascades that lead to ECM re-organization, removal of ECM debris, and clearance of apoptotic cells, thus favoring tissue healing. In this context, PTX3 is involved in the turnover of fibrin-rich deposits at wound sites after tissue injury, and consequent collagen deposition (16). Furthermore, when the stimuli persist or the resolution program is broken or stumbled, the inflammatory response may become chronic, impacting tissue remodeling and PTX3 expression. Macrophages are susceptible to the inflammatory environment and are key cells to modulate this system through PRMs. During the early inflammatory phase, M1‐polarized macrophages accumulate and orchestrate the inflammatory response. The subsequent switch to an M2-phenotype is crucial for resolving inflammation and tissue repair (17–19). M2-macrophages contribute to tissue homeostasis, dampening inflammation, scavenging ECM debris, and participating in tissue remodeling and repair (19, 20). On the other hand, apoptotic cells generated during chronic inflammation trigger the resolution, with significant changes in macrophage functions. Opsonization of apoptotic cells by PTX3 promotes their recognition by macrophages and subsequent efferocytosis (21), contributing to diverse M2-phenotypes switching (6) and regulating IL-10 and TGF-β1 production (22). Thus, PTX3 may play a homeostatic role in orchestrating tissue adaptation by coordinating leukocyte migration, resolution, and tissue healing.

PTX3 has been considered an essential regulator of airway mucosal surface homeostasis (23) and is useful as a disease or prognostic marker. In addition, the protein exerts a role in lung immunity against immunological dangers such as respiratory infections, allergy, tissue damage, and malfunction (24). As a humoral mediator of innate immunity, PTX3 opsonizes pulmonary pathogens promoting the clearance by phagocytosis and triggers the mucosal immune response to fungal or bacterial infections and respiratory viruses. Furthermore, PTX3 also has relevant roles in non-infectious pulmonary diseases. Altogether, PTX3 exerts multiple roles in respiratory diseases. However, its involvement in the development of IPF remains poorly explored.

## PTX3 and Lung Fibrosis: Lessons From Experimental Models of Pulmonary Fibrosis

**Figure 1:**
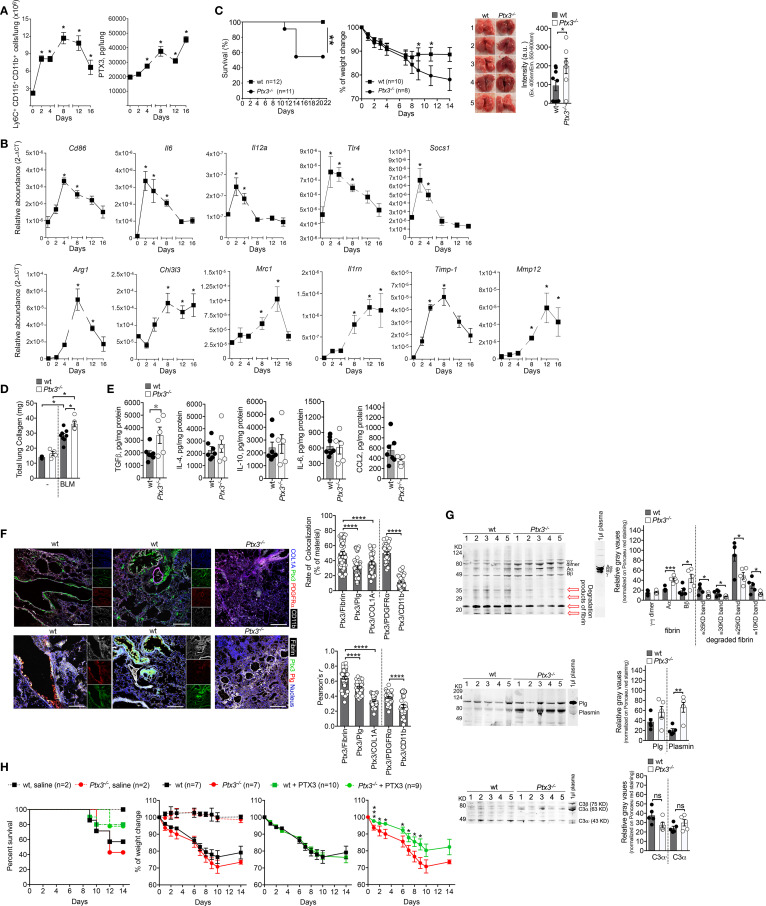
PTX3 protects mice from BLM-induced pulmonary fibrosis. A model of lung fibrosis was induced by BLM instillation (3.75 mg/Kg, *i.n.*) in Wild Type (WT) and *ptx3^-/-^* mice **(A-E)**. (**A**, left), kinetic of macrophage influx in the lungs of WT mice after BLM by FACS analysis. (**A**, right), kinetic of PTX3 lung content assessed by ELISA (PTX3 DuoSet® Kit ELISA DY2166, R&D Systems) in tissue homogenates. **(B)**, Transcription analysis of M1 genes (*Cd86-Mm00444543_m1*, *Il6-Mm99999064_m1, Il12a-Mm99999066_m1*, *Tlr4-Mm00445274_m1, Socs1-Mm00782550_s1*) and M2 genes (*Arg1-Mm00475988_m1, Chi3l3-Mm00657889_mH*, *Mrc1-Mm01329362_m1*, *Il1rn-Mm00446185_m1*, *Timp1-Mm00441818_m1*, *Mmp12-Mm00500554_m1*) markers of macrophage polarization by TaqMan probes (Applied Biosystems) at different days after BLM treatment. **(C, D)**, Susceptibility of *Ptx3^-/-^* mice to lung fibrosis induced by BLM instillation. (**C**, left) survival curve of mice (*100% of WT and 54.5% of Ptx3-/- mice), as* defined by humane end-points (e.g., weight loss of more than 25% of initial body weight, anorexia, excessive decrease in activity, shaggy hair, diarrhea, urinary retention, breathing difficulties). **P=0.01; Log-rank test. (**C**, middle) monitoring of weight loss. *P < 0.05; unpaired t-test). (**C**, right) representative photographs showing the appearance of lung parenchyma and quantification of autofluorescence intensity (excitation 405nm; emission collection at 550-60nm; CLARIOstar Microplate Reader, BMG Labtech) typically associated to hemoglobin in lung lysates of wt (n=9) and *Ptx3^-/-^* (n=7) mice (day 14). *P=0.05, unpaired t-test. **(D)** lung collagen content after 21 days assessed by Sircol assay. **(E)**, measurement of TGF-β_1_, IL-4, IL-10, IL-6, and CCL2 in lung lysates of wt (n=7) and *Ptx3^-/-^* (n=5) mice (day 14 after BLM treatment) by ELISA (R&D Systems). *P=0.05, unpaired t-test. **(F)**, confocal microscopy analysis on lung specimens (10µm) from WT mice (n=7) 14 days after BLM treatment. (**F**, upper panels) localization of PTX3 (green), Collagen I (blue), PDGFRα^+^ (red) mesenchymal cells and CD11b^+^ (white) immune cells. Representative localization of PTX3 around blood vessels (**F**, upper, left) or associated with fibrotic ECM and damaged epithelium (**F**, upper, middle). (**F**, lower panels) colocalization of PTX3 (green) with fibrin (white) and plasminogen (red) in fibrotic lung associated with blood vessels (**F**, lower, left) or ECM and damaged epithelium (**F**, lower, middle). Blue, nuclei. Lungs obtained from *Ptx3^-/-^* mice were used as control (Upper and lower panels, right). Bar, 100µm. The following antibodies were used: collagen I, rabbit polyclonal (5µg/ml; AbCam); PTX3, goat polyclonal (0.5µg/ml; R&D Systems); PDGFRα, BV421 rat (APA5, 1.5µg/ml; BD Horizon); CD11b, APC-Cy7™ rat (M1/70, 2µg/ml; BD Pharmingen); plasminogen, rat monoclonal (1µg/ml; Cell Sciences); fibrinogen, rabbit polyclonal (4µg/ml; Dako); species-specific Alexa Fluor 488/568/647- conjugated secondary antibodies were used. (**F**, right) Rate of colocalization (% of material; Fiji software) of PTX3 signal with fibrin, plasminogen, collagen I (COL1A), PDGFRα, and CD11b and relative Pearson’s correlation coefficient. Mean ± SEM of 5-8 images acquired for each mouse (n=7). *P < 0.0001, unpaired t-test. (**G**, upper), Western blot analysis of fibrin in lung lysates (10µg total proteins per lane on 10% SDS-PAGE) of WT (n=5) and *Ptx3^-/-^* (n=5) mice at day 7. A polyclonal rabbit anti-fibrinogen was used (3µg/ml; Agilent/DAKO). 1µl of basal mouse plasma in ACD-A (Anticoagulant Citrate Dextrose Solution) was used as a control for fibrinogen; 1µl of mouse plasma-ACD incubated with thrombin (1U/ml; 1h) was used as a control for fibrin. A typical band pattern of fibrin (Aα; Bβ, γ-γ dimer) is indicated in the fibrin control and lung lysates. Red arrows, lower molecular weight bands corresponding to degraded fibrin. (**G**, upper, right), quantification of fibrin bands as relative gray values (Fiji software) on Ponceau red staining. ***P < 0.005, *P < 0.5; unpaired t-test. (**G**, middle), Western blot analysis of plasminogen and relative band quantification as gray values (Fiji software) in same lysates (50µg total proteins per lane on 10% SDS-PAGE, **G**, middle, right). A polyclonal goat anti-plasminogen was used (0.5µg/ml; R&D Systems). The molecular weight of plasminogen and plasmin activation bands are indicated. (**G**, lower), Western blot analysis of the complement component C3 in the same lysates (10µg total proteins per lane on 10% SDS-PAGE) and relative band quantification as gray values (Fiji software, **G**, lower, right). A polyclonal goat anti-human/mouse C3 and activation fragments (1:3000; Merck-Millipore) was used. **(H)** Effect of PTX3 administration in BLM-induced lung fibrosis. One experiment was performed. Human recombinant PTX3 (50µg/mouse) was injected *i.p.* one day after BLM (5 mg/Kg, *i.n.*) treatment in WT mice. Survival (**H**, left) (*Ptx3-/- mice* from 42.8% to 77.8%, and WT mice from 57.1 to 80% of survival) and body weight (**H**, 3 panels right) were recorded until day 14. Curves referring to weight loss are shown compared to untreated WT and *Ptx3^-/-^* mice (**H**, first left) or separated by genotype and compared with the correspondent treated group (**H**, right). *P < 0.05; unpaired t-test. All results were expressed as mean ± SEM. Normalized data were analyzed by One-Way ANOVA with Tukey post-test, using the software GraphPad Prism 8.0. Differences were considered significant at P < 0.05.

Bleomycin (BLM) is vastly used to investigate the mechanisms involved in lung fibrosis in mice, and also in the selection of therapeutic drugs for IPF, including Pirfenidone and Nintedanib (25, 26). Several studies have addressed the relevance of PTX3 in different models of pulmonary fibrosis (27–29). Despite the observation that BLM induces PTX3 expression in murine models of lung fibrosis (27, 29), a paucity of information exists on the functional role of the protein in this model. It has been shown that PTX3 promotes murine fibrocyte differentiation dependent on FcγRI *in vitro* (28). *In vivo*, PTX3 is localized in fibrotic areas, and its distribution is associated with collagen deposition in lung parenchyma (28) and with macrophage infiltration at sites of fibrogenesis (29), revealing an interplay with macrophages during BLM-induced tissue fibrogenesis. Taking advantage of transgenic mice overexpressing PTX3 (Tie2-PTX3), it was recently shown that the protein could limit lung fibrosis, reducing collagen deposition and fibroblast activation and decreasing leukocyte recruitment (29). Using the BLM-induced fibrosis model (3.75 mg/Kg, *i.n*.), we confirmed macrophage (Ly6C^+^CD115^+^CD11b^+^) accumulation concomitantly with a progressive increase of PTX3 lung levels (**Figure 1A**), as previously described (30). Soon after BLM (2-4 days), RT-PCR showed an increased expression of M1-macrophage genes, followed later on (8-16 days) by an increased expression of M2-macrophage genes (**Figure 1B**). Therefore, increased PTX3 lung content is temporarily associated with a macrophage M2-polarization preceding the pulmonary fibrosis.

In the same model, *Ptx3^-/-^* mice showed reduced survival (**Figure 1C**) and accentuated weight loss (**Figure 1C**). Corroborating the results by Maccarinelli et al. (29), lungs from *Ptx3^-/-^* mice showed hemorrhagic areas (day-14)(**Figure 1C**) and increased fibrosis, as assessed by total collagen content (day-22)(**Figure 1D**). In *Ptx3^-/-^* mice lung homogenates (**Figure 1E**), fibrosis was not associated with differences in IL-4, IL-10, IL-6, and CCL2, thus indicating the independence of PTX3 in the regulation of inflammation (1, 2, 31), while we found increased TGF-β1 in *Ptx3^-/-^* mice (**Figure 1E**)(day-14). As described in several models of vascular pathology or tissue repair (16, 32), PTX3 controls the thrombotic response by influencing platelet activation and degranulation. Therefore, it is tempting to speculate that a specific increase in lung TGF-β1 may be due to a local release derived by platelet degranulation. PTX3 was found localized in the damaged alveolar epithelium and interstitial ECM associated with PDGFRα^+^ mesenchymal cells, and *Ptx3^-/-^* mice showed increased interstitial fibrin deposition and subsequent fibrotic scarring in ALI (acute lung injury) model (5). Similarly, in the BLM-induced fibrotic lungs (day-14), PTX3 is localized in damaged epithelium and areas of ECM rich in collagen-I, preferentially associated with PDGFRα^+^ mesenchymal cells rather than recruited CD11b^+^ cells (day-7)(**Figure 1F**). Moreover, PTX3 is localized around the blood vessels (**Figure 1F**). Thus, in line with evidence obtained in different experimental models of lung injury and repair (16, 33, 34), PTX3 plays a non-redundant and protective role during BLM-induced pulmonary fibrosis in mice.

PTX3 promotes arterial thrombosis (32) at wound sites during injury-induced thrombotic response and promotes healing by interacting with plasminogen, favoring timely fibrin removal in acidic microenvironments (5, 16, 33, 35). As presented in **Figure 1F**, in the BLM-induced fibrotic lungs PTX3 colocalized with fibrin deposits in ECM and damaged epithelium closely associated with plasminogen. A similar colocalization was observed in the endothelium of blood vessels possibly associated with coagulation sites (day-7)(**Figure 1F**). Coagulation proteases are recognized to exert pro-fibrotic cellular effects *via* activation of protease-activated receptors (PARs) (36). Fibrinolysis is also an essential prerequisite for subsequent tissue remodeling processes leading to efficient repair (37–40). Therefore, analysis of fibrin and plasminogen content in lung lysates would address whether a defective turnover in fibrin removal was present in *Ptx3^-/-^* mice. As assessed by Western blot, lungs of *Ptx3^-/-^* mice showed increased fibrin deposition and decreased fragments of fibrin degradation at the inflammatory phase (day-7)(**Figure 1G**). Differences in plasminogen deposition and plasmin formation (day-7)(**Figure 1G**) observed in the same lung homogenates suggest an impairment in ECM turnover of fibrin, possibly at the bases of a subsequent increased lung fibrosis. No evidence of PTX3 regulation on complement activation was observed in this model, as no differences were found in C3 deposition in the lung (**Figure 1G**). Several reports showed that coagulation cascade elements are involved in lung fibrosis (41). The deficiency of components of the fibrinolytic system caused exacerbated lung injury associated with defective clearance of necrotic tissue and augmented fibrin deposition and fibrosis (42). Lung fibrosis was reverted by overexpression of plasminogen activator genes (38, 39). Therefore, the disruption of fibrin removal and altered ECM turnover with collagen deposition appeared as the central mechanism underlying the phenotypes associated with *Ptx3^-/-^* mice in response to BLM, corroborating the observations from other models of organ damage (5, 33, 35). TGF-β1-mediated elevated PAI-1 levels and defective fibrinolysis have significant fibrotic consequences for tissue repair (43, 44). Interestingly, TGF-β1 was found to down-regulate PTX3 at mRNA and protein levels in granuloma (45) and mesenchymal cells (our results, data not shown). TGF-β1 increased ECM deposition promoting transcription of collagen and protease inhibitors, including tissue inhibitors of metalloproteases (TIMPs) and PAI-1 (46). Concomitantly, TGF-β1 decreases the secretion of proteases responsible for ECM degradation, including activators of plasminogen (43, 44), thus increasing the overall production of ECM proteins (47). Therefore, PTX3 down-regulation by TGF-β1 could inhibit the removal of the fibrin matrix and increases ECM deposition during experimental fibrosis.

The evidence obtained from the BLM-induced lung fibrosis model prompted us to evaluate the possible therapeutic effects of PTX3 treatment in the disease. In a first preliminary experiment, shown in **Figure 1H**, a single *i.p.* injection of recombinant PTX3 (50µg/mouse) one day after BLM (5 mg/Kg, *i.n.*) was sufficient to increase survival of *Ptx3^-/-^* mice, with a weak effect even in WT mice survival (**Figure 1H**). PTX3 treatment also attenuated the weight loss in *Ptx3^-/-^* mice but not in WT mice challenged with BLM (**Figure 1G**), suggesting a potential therapeutic effect of PTX3 in pulmonary fibrosis. However, the actual evaluation of potential PTX3 treatment requires more detailed pharmacological studies on the doses and administration routes.

## Mechanisms of PTX3 in Idiopathic Pulmonary Fibrosis: Interpreting Data From IPF

IPF is a chronic and lethal Interstitial Lung Disease characterized by fibroproliferation of unknown origin and insensitive to therapy, associated with excessive ECM deposition in the pulmonary parenchyma (48–50). Clinical signs include progressive loss of lung volume, increased respiratory effort, and abnormal gas exchange, leading to respiratory collapse (48–50). Currently, some hypotheses have been proposed about its controversial origin. Among them, clinical data have revealed a close correlation between pulmonary fibrosis and the profile of inflammatory mediators released by immune cells (19, 51, 52). Data supports that pulmonary fibrosis is the final result of previous alveolitis with excessive scarring (19, 52, 53). Current knowledge about IPF has been derived from detailed pathological analysis of human lung samples that elucidated its unique morphological characteristics, together with observations derived from animal models of disease (26, 52, 54, 55). New insights raised from genetic and transcriptomic studies on IPF samples have given a better comprehension of the molecular and cellular mechanisms determining the lung phenotype of IPF and patient therapeutics (25, 54, 56).

**Figure 2:**
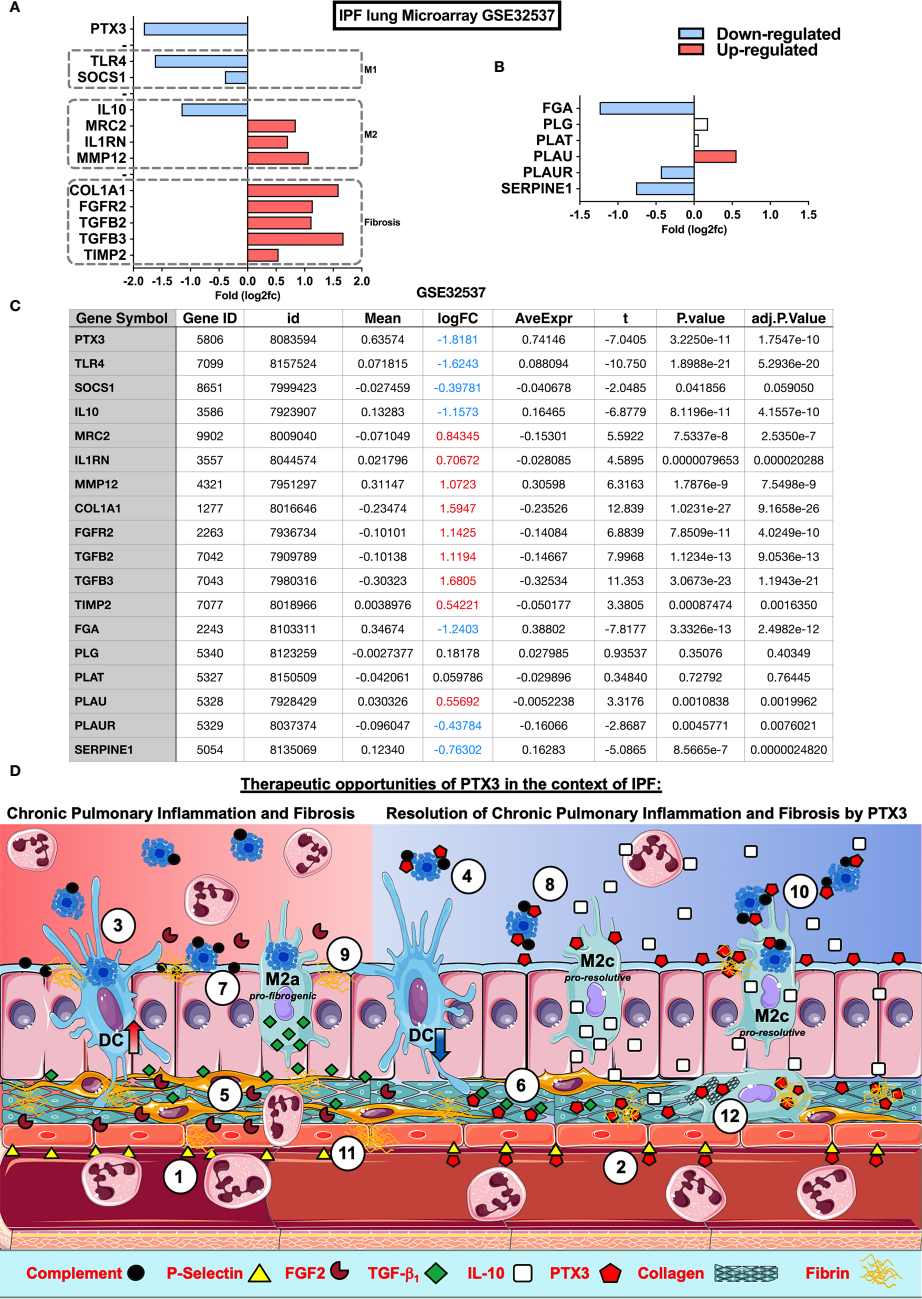
Impact of PTX3 in Idiopathic Pulmonary Fibrosis and therapeutic opportunities. Microarray analysis of lung samples from IPF (n=119) and healthy (n=50) individuals from GEO database: GSE32537 were analyzed by *Phantasus* (58) (https://genome.ifmo.ru/phantasus). **(A)** Lung expression of PTX3, fibrogenic markers as COL1A1, FGFR2, TGFB2, TGFB3 and TIMP-2, M1 (SOCS1 and TLR4) and M2 (IL-10, MRC2, IL1RN, and MMP-12) macrophage polarization markers **(B)** Expression of coagulation cascade FGA (Fibrinogen alpha-chain precursor), PLG (Plasminogen precursor), PLAT (Tissue-type Plasminogen Activator precursor), PLAU (Urokinase-type Plasminogen Activator precursor), PLAUR (Urokinase Plasminogen Activator Receptor) and SERPINE1 (PAI-1 Plasminogen Activator Inhibitor-1) in IPF and health samples. **(C)** Data table with analysis from GEO database GSE32537 analyzed by *Phantasus* according to the instructions for the use of the application. Differences were considered significant at *P value <0,05*. **(D)** Possible mechanisms and therapeutic opportunities of PTX3 in the context of IPF. IPF is characterized by a reduced PTX3 production (red background), however PTX3 may act as an anti-inflammatory as well as a pro-resolutive modulator of chronic pulmonary inflammation and fibrosis in IPF (blue background) at different levels: (1) Neutrophil influx is facilitated through the interaction with P-selectin expressed on the surface of ECs, (2) PTX3 could antagonize endothelial P-selectin, dampening neutrophil influx during chronic pulmonary inflammation; (3) the abundance of apoptotic cells in airways from IPF is related to DC phagocytosis and activation that sustain chronic lung inflammation, (4) PTX3 may block apoptotic cell internalization and consequent inflammation; (5) FGF2 activates fibroblasts and ECs, (6) however PTX3 interacts with FGF2 reducing its availability for binding to FGFR2 on fibroblasts and consequent fibrosis; (7) complement and apoptotic cell deposition in the lungs lead to chronic inflammation, (8) on the other side PTX3 may act as a scavenger preventing the excessive deposition of both complement components and apoptotic cells in lungs and consequent attenuation of tissue damage and inflammation; (9) Alveolar macrophages from IPF display defective efferocytosis and increased TGF-β1 production, contributing to tissue fibrogenesis, (10) while PTX3 may enhance macrophage efferocytosis and M2 polarization and resolution of inflammation by IL-10; (11) Finally, defective PTX3 production in IPF may increase fibrin deposition and fibrosis, (12) but PTX3 could contribute to the resolution of fibrosis, interacting with fibrin-clots and disorganized collagen fibers in the lung parenchyma, supporting fibrinolysis and clearance of ECM debris by macrophage phagocytosis, promoting lung tissue healing and repair.

The physiological role of PTX3 in IPF remains to be elucidated. However, present results and previous evidence suggest a role of PTX3 in lung repair in experimental models of lung injury (16), an association of PTX3 and Primary Graft Dysfunction (PGD) in IPF recipients after lung transplant (57), or a potential role of PTX3 produced by fibroblasts and bronchial epithelial cells in fibrocyte differentiation *in vitro* (28). In the lung tissue samples obtained from IPF patients, PTX3 was found associated with fibrotic areas of ECM, epithelium, and alveolar leukocytes (28). In order to gain a deeper insight into this association, we analyzed microarray data of lung samples from IPF (GEO database: GSE32537) using *Phantasus (* 58), a web application for visual and interactive gene expression analysis (https://genome.ifmo.ru/phantasus). Besides upregulated genes related to fibrosis (COL1A1, FGFR2, TIMP2, and TGF-β2/3; **Figures 2A, C**), increased expression of M2-macrophage and inhibition of M1-macrophage related genes were found in IPF samples (**Figures 2A, C**), similarly to WT mice exposed to BLM (**Figure 1B**). Moreover, IL-10 and PTX3 expression were down-regulated in IPF in this set of lung microarray (**Figure 2A**), as shown by normalized data (**Figure 2C**). PLAUR and SERPINE-1, genes belonging to the fibrinolytic system and related to tissue repair, were also down-regulated (**Figures 2B, C**). Thus, PTX3 gene down-regulation in lung samples may be related to active IPF pathology. This recapitulates the mouse phenotype observed *in vivo*, with aberrant collagen deposition in *Ptx3^-/-^* mice [**Figure 1** (29)], supporting the possibility that endogenous PTX3 exerts a protective role and may be involved in IPF disease. Therefore, PTX3 down-regulation could be part of a TGF-β1 regulatory program leading to fibrinolysis inhibition and ECM increased deposition, both determinants of IPF pathogenesis and progression.

## Therapeutic Opportunities of PTX3 in the Context of IPF: What Mechanisms Would Be Involved?

Data reported by Maccarinelli et al. (29) and our results suggest that IPF may be related to a low expression of PTX3 in lung tissue. Therefore, we could consider restoring PTX3 levels exogenously (as summarized in **Figure 1G**) as a possible therapeutic approach for IPF. Different roles of PTX3 could be considered:

## PTX3 as a Negative Modulator of Chronic Airway Inflammation in IPF

Chronic inflammation can lead to an imbalance in soluble factors production and leukocyte recruitment, turning the healing response into a pathological fibrotic response (52). Chronic neutrophilic airway inflammation occurs in IPF and airway neutrophilia, mainly due to CXCL8 produced by alveolar macrophages (59), and predicts mortality of IPF patient (60). PTX3 regulates neutrophils influx through interaction with P-selectin expressed on the surface of ECs (31, 61), thus, exogenous PTX3 may dampen neutrophil influx into IPF airways (**Figure 2D**). Moreover, soluble PTX3 derived from Human Umbilical Cord Blood-Derived Mesenchymal Stem Cells (UCB-MSCs) has anti-inflammatory effects in ALI, as shown in a model of neonatal hyperoxia-induced lung injury in rats. Similarly, adoptive transfer of MSC from WT but not from *Ptx3^-/-^* mice improved oxygenation with reduced lung collapse and neutrophils (33), shaping the differentiation of anti-inflammatory macrophages (62). On the other hand, Dendritic cells (DCs), activated by the phagocytosis of apoptotic cells, are described to sustain chronic lung inflammation in IPF (63). Nevertheless, in the presence of PTX3, DCs failed to internalize apoptotic cells (21), thus suggesting that PTX3 may prevent chronic pulmonary inflammation in IPF (**Figure 2D**). However, whether endogenous or exogenous PTX3 acts as a tissue-protective or resilience factor stimulating lung tissue reepithelization remains unexplored.

## PTX3 Involvement in Resolution of Inflammation in IPF

Efficient resolution of inflammation is crucial for the restoration of tissue integrity. PTX3 has been reported to induce the polarization of macrophages into anti-inflammatory M2 phenotype and to stimulate them to secrete the resolutive cytokine IL-10 (62, 64). Moreover, as mentioned above, PTX3 production by MSC reinforced M2 macrophage markers, inducing Dectin-1 and IL-10, and protecting mice from neonatal hyperoxia-induced lung injury (62). Therefore, PTX3 may contribute to the resolution of chronic inflammation in IPF *via* M2-macrophages enhancing IL-10-dependent anti-inflammatory and resolutive functions, such as neutrophil apoptosis (**Figure 2D**). Nevertheless, impaired efferocytosis can result in inflammation-associated pathologies (65). Indeed, efferocytosis by alveolar macrophages is impaired in IPF samples compared with other interstitial pneumonia (66), and a dysregulated or defective efferocytosis may contribute to the pathogenesis of IPF (65, 66). Notably, post-efferocytotic, satiated macrophages [also termed Mres (67)] produce high levels of TGF‐β1 (68). However, this production seems to be functionally antagonized by the production of IFN-β in these macrophages (69), that directs their anti-fibrotic phenotype (70, 71). Along these lines, apoptotic cell instillation after BLM attenuates lung injury (72) and induces PPARγ, promoting lung fibrosis resolution *via* regulation of efferocytosis and IL-10 production (73). In this way, PTX3 recognizes apoptotic cells and may facilitate the clearance of dead or dying cells (1). Therefore, the capacity of PTX3 to affect apoptotic cell recognition and efferocytosis could represent an additional mechanism of negative regulation of chronic inflammation in IPF (**Figure 2D**) (66). PTX3 enhanced complement-mediated clearance of apoptotic debris. The protein is recruited by C4 binding protein (C4BP) on apoptotic cells reducing the deposition of the lytic C5b-9 terminal complex at sites of tissue injury (74), limiting the complement-mediated tissue damage and inflammation (1), and possibly tissue fibrosis (**Figure 2D**).

## PTX3 as a Resolutive Modulator of Tissue Fibrosis in IPF

PTX3 interaction with plasminogen ensures the timely removal of fibrin deposits in the inflammatory ECM of the lung, allowing for the proper sequence of processes leading to efficient tissue repair. TGF-β1, the major fibrogenic molecule involved in the mechanisms of excessive ECM deposition in pulmonary fibrosis, negatively regulates PTX3 and ECM-degrading molecules (e.g., MMPs, uPA) and up-regulates TIMPs and PAI-1 (45, 75). Thus, defective PTX3-mediated fibrinolysis may represent a key mechanism underlying the development of the disease. Therefore, TGF-β1 induces suppression of PTX3-mediated fibrinolysis and may represent another mechanism underlying the development of IPF. In other contexts of tissue repair (16, 33, 35), PTX3 administration reversed the defective fibrinolysis associated with PTX3-deficiency and this may represent an important activity underlying PTX3 role in regulating the evolution towards fibrotic scarring of the lung (**Figure 2D**). PTX3 interacts with FGF2 and modulates the FGFR2-dependent vascularization of tumors and FGFR2-mediated smooth muscle cell proliferation and artery restenosis (76, 77). The expression of FGF2/FGFR2 axis is elevated in IPF samples (78), and FGFR2-dependent signaling is involved in pulmonary fibrosis (76, 79). Therefore, a down-regulation of PTX3 in IPF may represent a possible failure to antagonize these fibrotic pathways (**Figure 2D**). Finally, PTX3 also interacts with collagens and our own preliminary results indicate a possible involvement of collagen remodeling by mesenchymal cells, thus suggesting an effect in removing the excess of ECM from tissue parenchyma (**Figure 2D**) and hence in promoting tissue healing.

## Concluding and Remarks

Failure to control the overlapping events leading to the healing process is the cause of the functional tissue replacement by fibrous scar. Growing evidences indicate that abnormalities in pathways involving fibroblast activation and coagulation cascade drive abnormal fibroproliferation and progressive replacement of lung parenchyma by collagen (37, 80). Understanding the molecules involved in these pathways during aberrant wound repair may predict new targets and therapeutic intervention strategies. PTX3, besides being an essential fluid-phase PRM of the innate immune system, is involved in the healing at wound sites, favoring timely fibrinolysis through the interaction with fibrin and plasminogen. Results reported by Maccarinelli and colleagues and our observations indicate a protective and regulatory role of PTX3 in BLM-induced lung fibrosis models of lung injury. Besides IPF, other pathological conditions can result in pulmonary fibrosis, last but not least the persistent post-COVID syndrome (81, 82). Many questions remain open, starting from the therapeutic effect of PTX3 to the mechanisms involved in the protective role of the protein and the relationships of PTX3 with the homeostasis of the airway epithelium and with the collagen fibers of the ECM. Little is known about the effect of IPF therapy with Pirfenidone/Nintedanib on PTX3. Further clinical studies will be necessary to answer these and many other questions. Thus, in summary, we assessed the involvement of PTX3 in pulmonary fibrosis. Based on the literature and recent data, we propose that PTX3 may have a physiological and protective role during IPF, interacting with various circuits and representing a potential therapeutic target, acting as a pro-resolutive molecule in the context of pulmonary fibrosis.

## Data Availability Statement

The datasets presented in this study can be found in online repositories. The names of the repository/repositories and accession number(s) can be found below: https://www.ncbi.nlm.nih.gov/geo/, GSE32537.

## Ethics Statement

The animal study was reviewed and approved by by the Italian Ministry of Health (protocol approval n. 803/2015-PR).

## Author Contributions

AD, AM, BB, and RCR designed the discussion, conclusions and wrote the manuscript. All authors contributed to the article and approved the submitted version.

## Funding

RCR is grateful to the Conselho Nacional de Desenvolvimento Científico e Tecnológico (CNPq, Brazil) under Grant Agreement No. 312839/2020-0. AD, BB, and AM are grateful to the Italian Association for Cancer Research (AIRC; grant number IG- 23465) and the Italian Space Agency (ASI MARS-PRE Project, grant number DC-VUM 2017-006) for financial support.

## Conflict of Interest

AM and BB are inventors of patents on pentraxin-3 and obtain royalties on related reagents.

The remaining authors declare that the research was conducted in the absence of any commercial or financial relationships that could be construed as a potential conflict of interest.
